# Synthetic anti-angiogenic genomic therapeutics for treatment of neovascular age-related macular degeneration

**DOI:** 10.1016/j.ajps.2021.04.001

**Published:** 2021-05-07

**Authors:** Jing Wang, Xiang Shi, Qiyu Bo, Hong Wang, Fang Wei, Jun Liu, Hao Wang, Liuwei Zhang, Yan Qi, Zhen Li, Qixian Chen, Xiaodong Sun

**Affiliations:** a Department of Ophthalmology, Shanghai General Hospital, Shanghai Jiao Tong University School of Medicine, Shanghai 200080, China; b Shanghai Key Laboratory of Ocular Fundus Diseases, Shanghai General Hospital, Shanghai Jiao Tong University School of Medicine, Shanghai 200080, China; c Shanghai Engineering Center for Visual Science and Photomedicine, Shanghai General Hospital, Shanghai Jiao Tong University School of Medicine, Shanghai 200080, China; d National Clinical Research Center for Eye Diseases, Shanghai General Hospital, Shanghai Jiao Tong University School of Medicine, Shanghai 200080, China; e Ningbo Hygeia Medical Technology Co., Ltd., Ningbo 315201, China; f School of Bioengineering, Dalian University of Technology, Dalian 116024, China; g College of Pharmacy, Dalian Medical University, Dalian 116044, China

**Keywords:** Age-related macular degeneration, Anti-angiogenesis, Gene therapy, Polymer, Vascular endothelial growth factor

## Abstract

In light of the intriguing potential of anti-angiogenic approach in suppressing choroidal neovascularization, we attempted to elaborate synthetic gene delivery systems encapsulating anti-angiogenic plasmid DNA as alternatives of clinical antibody-based therapeutics. Herein, block copolymer of cyclic Arg-Gly-Asp-poly(ethylene glycol)-poly(lysine-thiol) [RGD-PEG-PLys(thiol)] with multifunctional components was tailored in manufacture of core-shell DNA delivery nanoparticulates. Note that the polycationic PLys segments were electrostatically complexed with anionic plasmid DNA into nanoscaled core, and the tethered biocompatible PEG segments presented as the spatial shell (minimizing non-specific reactions in biological milieu). Furthermore, the aforementioned self-assembly was introduced with redox-responsive disulfide crosslinking due to the thiol coupling. Hence, reversible stabilities, namely stable in extracellular milieu but susceptible to disassemble for liberation of the DNA payloads in intracellular reducing microenvironment, were verified to facilitate transcellular gene transportation. In addition, RGD was installed onto the surface of the proposed self-assemblies with aim of targeted accumulation and internalization into angiogenic endothelial cells given that RGD receptors were specifically overexpressed on their cytomembrane surface. The proposed anti-angiogenic DNA therapeutics were validated to exert efficient expression of anti-angiogenic proteins in endothelial cells and elicit potent inhibition of ocular neovasculature post intravitreous administration. Hence, the present study approved the potential of gene therapy in treatment of choroidal neovascularization. In light of sustainable gene expression properties of DNA therapeutics, our proposed synthetic gene delivery system inspired prosperous potentials in long-term treatment of choroidal neovascularization, which should be emphasized to develop further towards clinical translations.

## Graphical abstract

**Figure:**
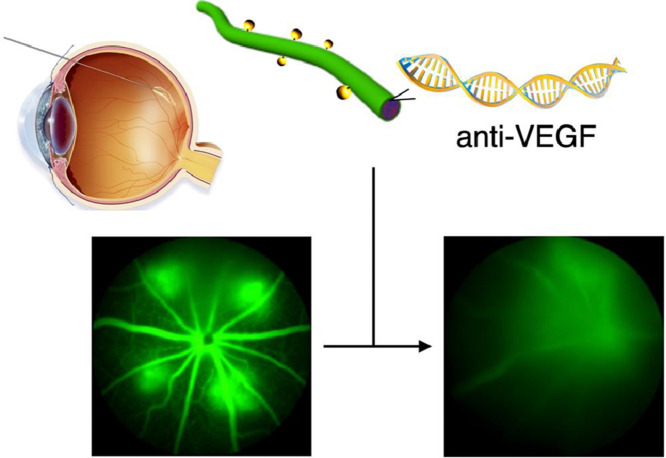

## 1 Introduction

Indulgence in electronic displays, owing to their high-energy visible light and ultraviolet, has prompted a rapidly growing population of choroidal neovascularization (CNV), which has been acknowledged to be a major cause of blindness across the world. In reference to the recent statistics, CNV comprises an estimated 2.5% of people aged over 40 years [[1], [2]–3]. Note that wet age-related macular degeneration (wAMD) was characterized by CNV, which provokes progressive leakage of blood and fluid, eventually leading to vision loss [[4], [5]–6]. In principle, CNV is stimulated by vascular endothelial growth factor (VEGF), diffusible cytokines that prompt angiogenesis and vascular permeability [7,8]. Pertaining to the clinical therapeutic regime, intravitreal administration of VEGF antagonists (e.g. ranibizumab or bevacizumab) that specifically bind to the VEGF, have been verified to be a valid approach in suppressing CNV progression of wAMD and improving visual acuity [[9], [10], [11], [12], [13]–14]. Nonetheless, the aforementioned antibody-based therapeutics are required to be repeatedly administered at 4–8 weeks interval, which not only conflicts the patient compliance but also increases the risks of ocular inflammation, retinal injury and endophthalmitis [15,16]. To this drawback, gene therapy as alternative to antibody-based therapeutics is desirable in light of long-term protein expression of the encoded therapeutic gene in the affected cells [17,18]. For instance, genomic therapeutics recently developed by our laboratory have demonstrated intriguingly consistent gene expression in cell spheroids over 90 days, and the levels of the expressed proteins were recorded to be appreciably constant [19]. Hence, this tempting constant gene expression profile is postulated to not only allow reduced dosage frequencies (thus improved patient compliance) but also contribute to improved therapeutic outcomes in respect to the constant retention of high level of therapeutic proteins in the pathologic sites.

However, to accomplish gene therapy, appropriate delivery systems are imperative to transport the inherently biologically fragile DNA to the cell interior (cell nucleus) for efficient gene expression since the supramolecular DNA owing to its large scale (micrometer) and intrinsic negative-charge forbids its diffusion across the lipophilic and negatively charged cytomembranes, not to mention the other biological barriers hindering the successful intracellular gene transportation [20,21]. It should be noted for viral vectors, despite their tempting transfection efficiencies, whose fatal concerns (e.g. severe immunogenicity, limited gene-loading capacity and difficulties in industrial scale-up) motivate us to seek an alternative approach in manufacture of delivery vehicles for intracellular gene transportation [22,23]. To the essential prerequisites for gene delivery, we intended to exploit materials-based molecular design in manufacture of non-viral synthetic gene delivery systems to accomplish gene therapy.

In the present research, the negatively charged plasmid DNA was schemed to electrostatically complex with cationic block copolymers of poly(ethylene glycol)-polylysine (PEG-PLys). Consequently, the DNA condensates initialized by electrostatic complexation with the cationic PLys segments as the internal core, was postulated to be surrounded by the hydrophilic and biocompatible PEG shell. With the aim of adequate colloidal stability in the physiological environment, thiol groups were introduced into the side chains of PLys with the intention of crosslinking of the internal pDNA/PLys core by means of disulfide linkage between the thiols. Notably, the disulfide linkage was fairly stable in the extracellular milieu (relative oxidizing environments), thereby affording substantial stability to withstand the potential structural dissociation in the bloodstream or extracellular milieu. On the contrary, the disulfide bond is susceptible to cleavage in the intracellular compartments. Note that the intracellular milieu is characterized to be remarkably reducing compartments, intracellular structural disassembly from the proposed self-assemblies could be readily anticipated in subsequence to disulfide cleavage, which consequently facilitates the pDNA release from the aforementioned DNA self-assemblies for smooth gene expression machinery. On the other hand, targeting moieties of cyclic Arg-Gly-Asp (RGD) peptide were also installed onto the surface of the PEGylated self-assemblies. In view the specific affinities of RGD peptide to the angiogenic vasculatures (the receptors of RGD, including α_V_β_3_ and α_V_β_5_ integrins, are overexpressed on the angiogenic endothelial cells), hence preferential accumulation and thus efficient protein expression of therapeutic proteins (anti-VEGF) in CNV could be envisioned in the treatment of wAMD.

## 2 Materials and methods

### 2.1 Materials

*α*-Methoxy-*ω*-amino-PEG (M_w_ 12,000) and acetal-PEG-NH_2_ (M_w_ 12,000) was purchased from SinoPEG Co. (Xiamen, China). N-Trifluoroacetyl-L-lysine N-carboxyanhydride [Lys(TFA)-NCA] was purchased from Hubei Jusheng Co. (Wuhan, China). Dithiothreitol (DTT), diethylenetriamine (DET), N,N-dimethylformamide (DMF), dichloromethane, benzene, and trifluoroacetic acid were purchased from Wako Pure Chemical Industries, Ltd. N-succinimidyl-3-(2-pyridyldithiol)propionate (SPDP) was purchased from Santa Cruz Biotechnology, Inc. (Santa Cruz, CA). Fetal bovine serum (FBS) was purchased from Dainippon Sumitomo Parma Co., Ltd. (Osaka, Japan). Cell culture lysis buffer and luciferase Assay System Kit was purchased from Promega Co. (Madison, WI). Dulbecco's modified Eagle's medium (DMEM) was purchased from Sigma–Aldrich (St. Louis, MO). In pertinent to the cellular uptake and intracellular distribution assay, pDNA was labeled with Cy5 using a Label IT Nucleic Acid Labeling Kit from Mirus Bio Corporation (Madison, WI) according to the manufacturer's protocol. For aqueous phase SEC, LC-2000 system (JASCO, Tokyo, Japan) equipped with Superdex200 10/300 GL (GE Healthcare, Tokyo, Japan) and a UV detector was used for characterizations. All animal experimental procedures were performed in accordance with the Guide for the Care and Use of Laboratory Animals as stated by the guidelines of Shanghai General Hospital (Shanghai Jiao Tong University School of Medicine).

### 2.2 Polymer synthesis

Block copolymer of PEG-PLys(thiol) and RGD-PEG-PLys(thiol) was synthesized based on ring opening polymerization. In brief, amine-functionalized Meo-PEG-NH_2_ was employed as macroinitiator for polymerization of monomers of Lys(TFA)-NCA for block copolymeric PEG-PLys(TFA). The protective TFA groups was removed by hydrolysis reaction in NaOH. Furthermore, thiol groups were introduced into the side chain of the synthetic PLys segment in block copolymeric PEG-PLys based on its reaction with SPDP. The yielded PEG-PLys(SPDP) was transferred under treatment of DTT to obtain PEG-PLys(thiol). On the other hand, acetal-PEG-NH_2_ was employed for synthesis of acetal-PEG-PLys(thiol) by following the similar synthetic route, which was transferred for acidic treatment to yield aldehyde-PEG-PLys(thiol). Cysteine-functionalized cyclic RGD was attempted for reaction with aldehyde group of aldehyde-PEG-PLys(thiol) to obtain the ultimate RGD-PEG-PLys(thiol). The detailed synthetic procedures were described in Supporting Information.

### 2.3 Dynamic light scattering (DLS)

The hydrodynamic diameters of the formulated pDNA delivery systems were measured by DLS (Zetasizer nanoseries, Malvern Instruments Ltd., Worchestershire, UK). All DLS measurements were conducted independently for at least three times at 25 °C. Note that the rate of decay in the photon correlation function was analyzed by referring to a cumulant method. Accordingly, the yielded hydrodynamic diameters were concluded based on the Stokes-Einstein equation.

### 2.4 Cellular uptake

Human umbilical vein endothelial cells (HUVECs) were seeded onto 6-well culture plates (50 000 cells/well) in cell culture medium (2 ml) containing 10% FBS and 1% antibiotics (penicillin and streptomycin) and incubated in a humidified atmosphere supplemented with 5% CO_2_ at 37 °C. After 24 h incubation, the medium was replaced with fresh medium. The cells were treated by addition of PEG-PLys/pDNA, PEG-PLys(thiol)/pDNA and RDG-PEG-PLys(thiol)/pDNA (1 µg of Cy5-labeled pDNA/well) and followed by another 24 h incubation. The medium was discarded, and the cells were washed three times with ice-cold PBS to remove extracellular fluorescence. Furthermore, the cells were subjected to trypsin-EDTA treatment, and the trypsinized cells were harvested and resuspended in 1 ml ice-cold PBS. the cell suspension was filtered through a nylon mesh and transferred for analysis by a BD™ LSR II flow cytometer equipped with FACS-Diva™ software (BD Biosciences, Franklin Lakes, NJ) to quantify the intracellular pDNA quantity. Note that the cellular uptake efficiencies were expressed as the mean fluorescence intensities from Cy5-pDNA based on three independent samples (*n* = 3).

### 2.5 Gene expression efficiency

HUVECs were seeded onto 24-well culture plates (20 000 cells/well) in 400 µl cell culture medium containing 10% FBS and 1% antibiotics (penicillin and streptomycin) and incubated in a humidified atmosphere supplemented with 5% CO_2_ at 37 °C. At 24 h post incubation, gene delivery systems containing the reporter pDNA od CAG-Luc were added to each well for 48 h incubation. Note that fresh medium was replaced at 24 h interval. At 48 h post transfection, the cells were washed three times with 400 µl ice-cold PBS and lysed in 150 µl cell lysis buffer at 37 °C for 10 min. Furthermore, 20 µl of the cell lysate was transferred into a 96-well luminometry plate, followed by addition of 100 µl of Luciferase Assay Reagent (Promega, Madison, WI), and allowed for 15 min reaction. The expressed Luc protein was quantified for 10 s recording of the photoluminescence intensities by Mithras LB 940 (Berthold Technologies, Bad Wildbad, Germany). Meanwhile, the quantities of the total protein in the cell lysate was quantified according to the classical Micro BCA™ Protein Assay Kit (Pierce, Rockford, IL). The obtained Luc activities was normalized against the corresponding quantities of the total protein in the cell lysates. Herein, the gene expression levels were expressed as relative light units (RLU) per mg of protein (RLU/mg protein) (*n* = 4).

### 2.6 Animals

C57BL/6 mice (approximate 7 weeks, 20 g) were used in anti-CNV study. Note that all the animal experiments were conducted strictly according to the guidelines of the ARVO statement for the Use of Animals in Ophthalmic and Vision Research. The experimental protocols were approved by Institutional Animal Care and Use Committee of Shanghai Jiao Tong University (Shanghai, China). The mice were housed and subjected to containment in the animal care services facility with 12 h light/dark cycle and constant access to nourishments.

### 2.7 Laser-induced CNV

CNV was established according to our previously reported protocol. In brief, pupil dilatation was performed with tropicamide (Santen, Osaka, Japan), followed by anesthetization based on intraperitoneal injection of 1% pentobarbital sodium (0.1 ml/10 g body weight, Guge Biotech, Wuhan, China) into C57BL/6 mice. To proceed photoagulation, loxacin eye ointment (Xing Qi Pharmaceutical Companies, Shenyang, China) was placed on top of corneal, and four laser spots were located at the vicinities of the optic nerve head with an argon laser (110 mW, 100 ms, 50 µm, OcuLight Infrared Laser System 810 nm, Iridex Corp., Mountain View, CA, USA). Appearance of a gray bubble indicative of the rupture of Bruch's membrane were included. If retinal bleeding occurred, the animal was eliminated. Eyes were enucleated at different time points.

### 2.8 Intravitreal injection

Synthetic gene delivery systems containing sFlt-1 were intravitreal administered into the vitreous cavity using fine, 32-G needles and 10 µl syringes (cat. No 80,330; both form Hamilton, Reno, NV). Note that the injection process was carefully conducted with the aids of microscope through the flattened cornea (covered with ointment), and the tip of the needle is required to penetrate the sclera, choroid, and retina to reach the vitreous cavity. An approximate volume of 2 µl per injection was applied to each eye.

### 2.9 Fluorescence angiography

FA imaging was conducted on day 7 post photocoagulation to assess the severity of CNV leakage. Prior to FA imaging, mice were subjected to sequential intraperitoneal dosage of 1% sodium pentobarbital (Guge Biotech, Wuhan, China) for anesthetization at 5 µl/g body weight and 10% fluorescein sodium (0.5 ml, Fluorescite, Alcon, Tokyo, Japan) for bio-imaging. Fundus angiogram photos were captured at the middle stage (2–3 min post injection of fluorescein sodium) using a digital fundus camera (Heidelberg Retina Angiograph, Vista, CA). Furthermore, the relative fluorescence intensity of each CNV lesion was quantified with the aids of ImageJ platform (National Institutes of Health, Bethesda, MD). The average intensity of each CNV lesion with leakage was calculated according to our previously reported protocol (Burgess et al., 2010). In brief, an outline was drawn to encompass each CNV area, and mean fluorescence was measured, along with three adjacent background readings. The total corrected CNV fluorescence (TCCF) = integrated density – (area of selected area × mean fluorescence of background readings), was calculated. This TCCF was equalized against the mean TCCF of retina vessels, with results presented as fold increase over interphase levels. We defined the intensity of retinal vessels as one and the avascular area as zero and the signal intensity for each pixel was represented from zero (darkest) to one (brightest).

### 2.10 Perfusion fixation

Immunofluorescence procedures require perfusion fixation to stabilize the proteins or peptides in the choroid in order to allow for subsequent antibody binding to the antigenic site on those proteins or peptides. At end points of Day 7 after laser and sFlt-1 treatment, mice were perfused transcardially with cold 4% paraformaldehyde using a previously described protocol with minor modifications (Gerfen, 2003). In brief, mice were administered with an over dose (0.2 ml) of 1% sodium pentobarbital and monitored until the point when the animal fails to respond to pinching of the foot. Incisions in the abdomen and diaphragm were made to expose the heart and perfusion needle was placed into ascending aorta. Cold 4% paraformaldehyde was poured into left ventricle of mouse through a peristaltic perfusion pump (Cole-Parmer Masterflex, NY). Twitching of muscles suggests that the perfusion is proceeding properly. After the effluent runs clear, pump was stopped, eyes and spleens were harvested, post-fixed for 2 h and then placed in PBS to make choroidal flatmounts or put into 30% sucrose solutions to make frozen sections.

### 2.11 Choroidal flatmount and immunofluorescence staining

Choroidal flatmounts were performed under an operating microscope (Olympus, Tokyo, Japan) subsequent to perfusion fixation and post-fixation. In brief, the cornea, lens and vitreous were removed from the harvested eye. The retina was carefully dissected from the choroid. Furthermore, six radial incisions were applied to the remaining RPE-choroid cup for flattened choroid, which is immediately transferred for immunostaining treatment. Prior to immunostaining, the RPE-choroid complexes were blocked in 5% goat serum albumin (containing 0.3% Triton X-100) for 1 h at room temperature, followed by incubation with FITC-labeled isolectin-B4 (IB4) (FL-1201, Vector Laboratories, Burlingame, CA) at 4 °C. After overnight reaction, the RPE-choroid complexes were rinsed and transferred for incubation with Alexa Fluor 488-labelled secondary antibodies (SA00003-11, Proteintech, Chicago, MI) for 1 h at room temperature. Eventually, fluorescence images were conducted under a fluorescence microscope (Olympus, Tokyo, Japan) or a confocal laser scanning microscope (CLSM) (Leica TCS SP8, Leica TCS NT, Wetzlar, Germany).

### 2.12 Statistical analysis

The *P* values were determined based on the Student's *t*-test using a two-tailed distribution and two-sample un-equal variance with the *T*. Test function of Microsoft Excel. The *P* values less than 0.05 were considered as statistically significant.

## 3 Results and discussion

### 3.1 Synthesis of block copolymeric RGD-PEG-PLys(thiol) and chemical characterizations

**Fig. 1:**
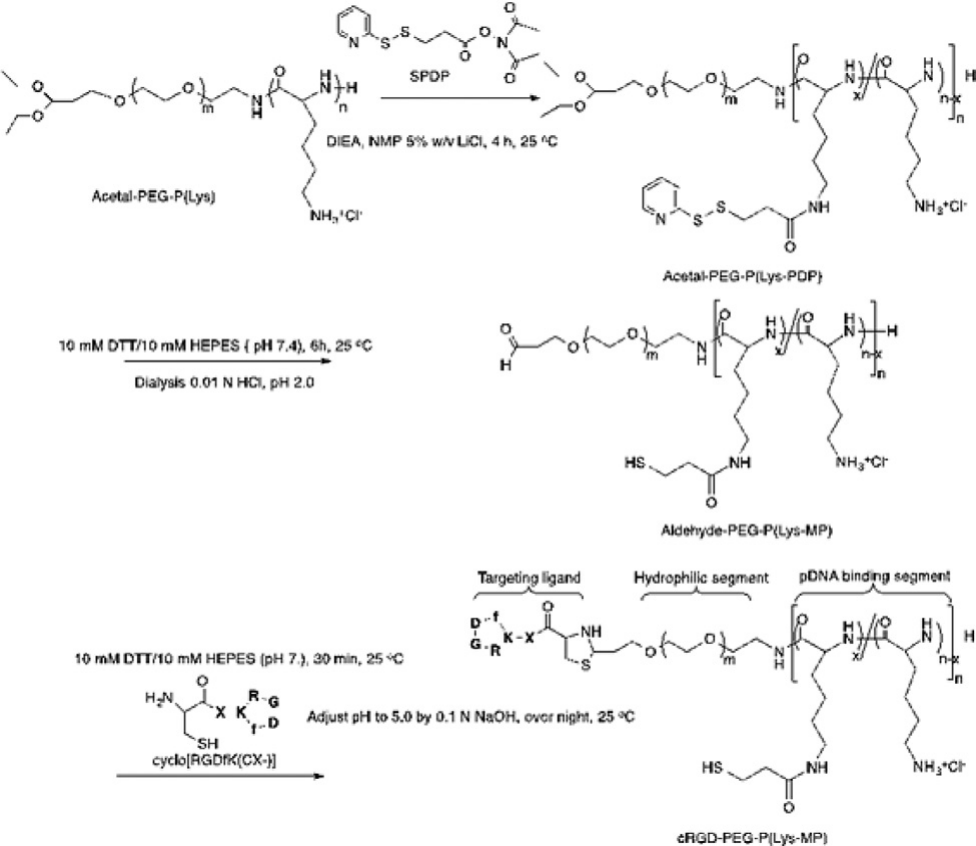
Synthetic route in synthesis of block copolymeric RGD-PEG-PLys(thiol).

The block copolymer of RGD-PEG-PLys(thiol) was synthesized according to Fig. 1. In brief, acetal-PEG-NH_2_ was employed as the macro-initiator for ring-opening polymerization of the monomers of Lys(TFA)-NCA for production of acetal-PEG-PLys(TFA). Furthermore, the protecting TFA residues in the units of PLys(TFA) segment were removed under treatment in NaOH (0.1 N) to yield cationic bloc copolymer of acetal-PEG-PLys. Furthermore, the amino groups of lysine in acetal-PEG-PLys were used for conjugation of SPDP, followed by DTT treatment to yield the thiol groups. Ultimately, acetal-PEG-PLys(thiol) was incubated in acidic condition for transformation of the terminus acetal groups into aldehyde groups, which was schemed to react with cysteine groups from RGD to yield the ultimate block copolymer of RGD-PEG-PLys(thiol). The resulting copolymeric RGD-PEG-PLys(thiol), as well as the control copolymers of PEG-PLys, were characterized by both ^1^H NMR and C-NMR measurements (Figs. S1–S5). As shown in Fig. S5, the polymerization degree of PLys segment was determined to be approximate 60.4, and the percentage of thiol substitution was estimated to be approximate 14.6% in relative to the total lysine units. Moreover, successful conjugation of cyclic RGD to the block copolymer was verified due to the evident peaks at 7.2–7.3 ppm, assignable to the protons from the phenyl groups of RGD, and the conjugation efficiency was estimated to exceed 97.2% per block copolymer. GPC measurement for the ultimate RGD-PEG-PLys(thiol) copolymer verified appreciable unimodal molecular weight distribution (Fig. S6).

### 3.2 Fabrication of gene delivery systems based on electrostatic self-assembly and physiochemical characterizations

**Fig. 2:**
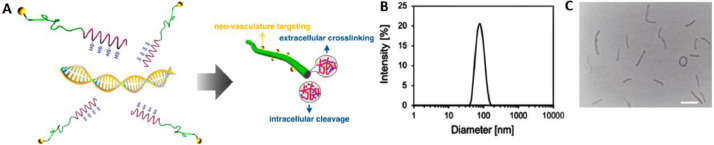
Schematic illustration and physiochemical characterizations of synthetic gene delivery system from RGD-PEG-PLys(thiol). (A) Illustration in manufacture of multifunctional genomic nanoconstructs from self-assembly of RGD-PEG-PLys(thiol) and pDNA; (B) DLS (C):TEM for insight on the morphologies of RGD-PEG-PLys(thiol)/pDNA. Scale bar: 100 nm.

The yielded functional block copolymer of RGD-PEG-PLys(thiol) was used to complex with polyanionic pDNA under gentle vortex, followed by 5 min incubation and sequential dialysis against DMSO-containing aqueous solution (DMSO: 10 mM) and distilled water for disulfide crosslinking. Note that the positively charged PLys segments could electrostatically assemble with the negatively charged plasmid DNA (pDNA) into polyionic complex core, and the non-ionic hydrophilic PEG segments would surround the formed PLys/pDNA complex core as the external shells (Fig. 2A). The resulting formation of RGD-PEG-PLys(thiol)/pDNA was characterized by DLS. As shown in Fig. 2B, nanoscaled formation possessing approximate 61.2 nm in hydrodynamic diameter was confirmed with unimodal size distribution (PDI: 0.09), which is contrast to the larger scale of naked pDNA (approximate 700 nm in hydrodynamic diameter by DLS measurement), suggesting the electrostatic condensation of pDNA by polycationic RGD-PEG-PLys(thiol) into favorable sub-100 nm nanoscale structures. Meanwhile, neutralization of the negative charges of pDNA (−38.4 mV) was also verified post complexation with RGD-PEG-PLys(thiol), as evidenced by the appreciable +2.4 mV for RGD-PEG-PLys(thiol)/pDNA. This appreciable neutral surface charge verified the excellent charge-masking function from PEG surface modification, which is critical in reducing electrostatic reactions in the complicated biological environment. Charge neutralization of pDNA was also confirmed by gel electrophoresis study for pDNA upon complexation with RGD-PEG-PLys(thiol)/pDNA at varied *N*/*P* ratios (defined as the molar ratio of the amine groups from block polymer to the phosphate groups from pDNA). As shown in Fig. S7, complete complexation of pDNA was identified at *N*/*P* ratios exceeding stochiometric charge ratio (*N*/*P* ≥ 1). Furthermore, the morphologies of pDNA condensates were characterized by TEM measurement. As shown in Fig. 2C, distinctive nanoscaled rod-like structures were observed, whose average length was measured to be approximate 100 nm. This distinctive rod-shape condensate is not surprising with respect that double-strand DNA (dsDNA) is rigid macromolecule, whose persistence length is approximate 50 nm in physiological condition. Consequently, the condensation of pDNA would follow a regular folding scheme into DNA bundles rather than collapse into spherical structures [[24], [25]–26]. Of note, the obtained rod-shaped nanostructures were reported to exert appreciable high cellular uptake activities, as well as high gene expression activities due to the facilitated transcription by means of the regular DNA folding scheme [26].

### 3.3 Reversible stabilities of gene delivery systems due to redox crosslinking

**Fig. 3:**
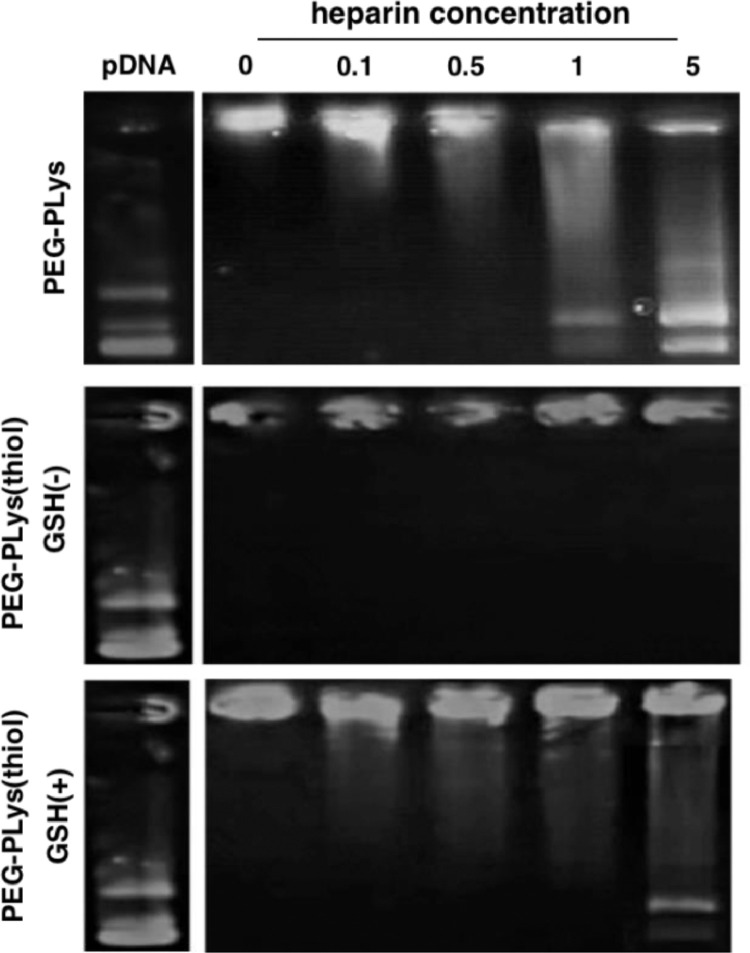
Insight on structural dissociation of pDNA self-assemblies from PEG-PLys and PEG-PLys(thiol) under incubation with polyanionic heparin at varied concentrations (units: mg/ml).

Aiming for adequate colloidal stabilities in the physiological environment, the aforementioned self-assemblies were introduced with redox-responsive crosslinking so as to withstand the potential exchange reactions and thus prevent the premature dissociation in the harsh physiological environment. As shown in gel electrophoresis (Fig. 3), ready structural dissociation and thus liberation of the pDNA payloads was confirmed for PEG-PLys/pDNA self-assemblies (void of disulfide crosslinking) in presence of bio-existing polyanionic glycosaminoglycan (heparin: 1.0 mg/ml). In contrast, the colloidal stabilities were observed to be substantially improved for PEG-PLys(thiol)/pDNA self-assemblies as evidenced by negligible pDNA liberation despite their incubation in presence of polyanionic heparin at a concentration of 5.0 mg/ml (Fig. 3). This observation approved the functional role of disulfide crosslinking in strengthening colloidal stabilities of electrostatically-based self-assemblies in the biological milieu. Aside from adequate colloidal stabilities in the extracellular milieu, the proposed RGD-PEG-PLys(thiol)/pDNA self-assemblies is also designed to be capable of readily liberating the pDNA payloads in the cell interiors so as to facilitate the subsequent transcription and translation machinery. Given that the cell interior is characterized to be a significantly reducing compartment that commits to elimination of the detrimental reactive oxygen species, for instance, abundant reducing substances of glutathione (GSH) have documented in the cytosol (in the range of several 10–100 mM), yet the extracellular level of GSH was estimated to be in the range of serval 10–100 µM [27,28]. Therefore, the redox-responsive disulfide linkage in the constructed nanocolloidals is envisioned to be selectively cleavable in the cytosol, while the disulfide crosslinking is fairly stable in extracellular milieu (including extracellular matrix and bloodstream). Consequently, the electrostatically-assembled nanocolloidals lack of chemical crosslinking are susceptible to exchange reactions with polyanionic species in view of diverse anionic polyanions existing in the intracellular compartment (e.g. nucleic acids) and on the cytoplasm structures (e.g. glycosaminoglycans), leadingly facilitating pDNA release for subsequent DNA transcription and translation machinery. In consistent with our speculation, readily pDNA release was confirmed for RGD-PEG-PLys(thiol)/pDNA self-assemblies in presence of GSH (50 mM, mimicking the intracellular milieu), comparable to PEG-PLys/pDNA. To this end, the facile redox-responsive stabilities were confirmed for the proposed RGD-PEG-PLys(thiol)/pDNA self-assemblies, which afford adequate colloidal stabilities in the extracellular environment but readily dissociation and release of pDNA payloads in the targeted cell interiors, thereby beneficial in accomplishing promoted gene expression in the targeted cells.

### 3.4 Promoted cellular uptake and transfection due to disulfide crosslinking and surface RGD modification

**Fig. 4:**
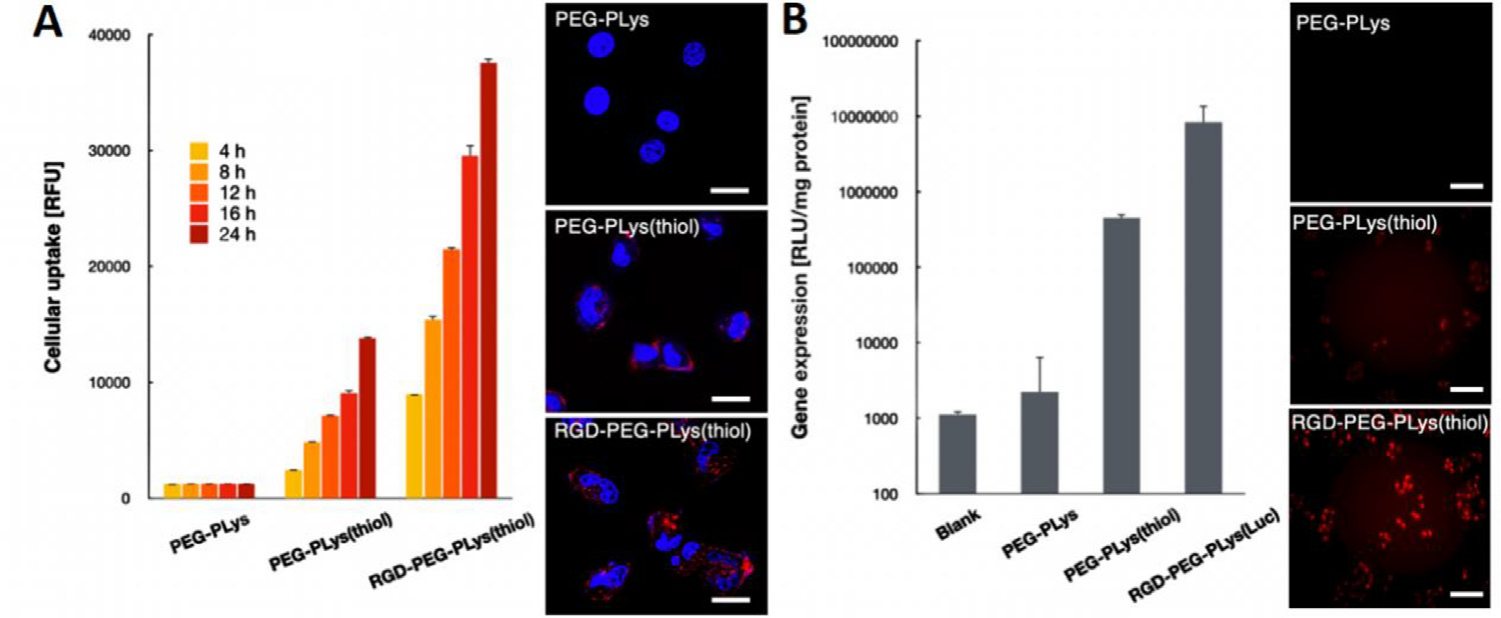
*In vitro* performance of a class of synthetic gene delivery systems upon incubation in HUVECs. (A) cellular uptake efficiencies, scale bar: 20 µm; (B) gene expression efficiencies, scale bar: 100 µm.

**Fig. 5:**
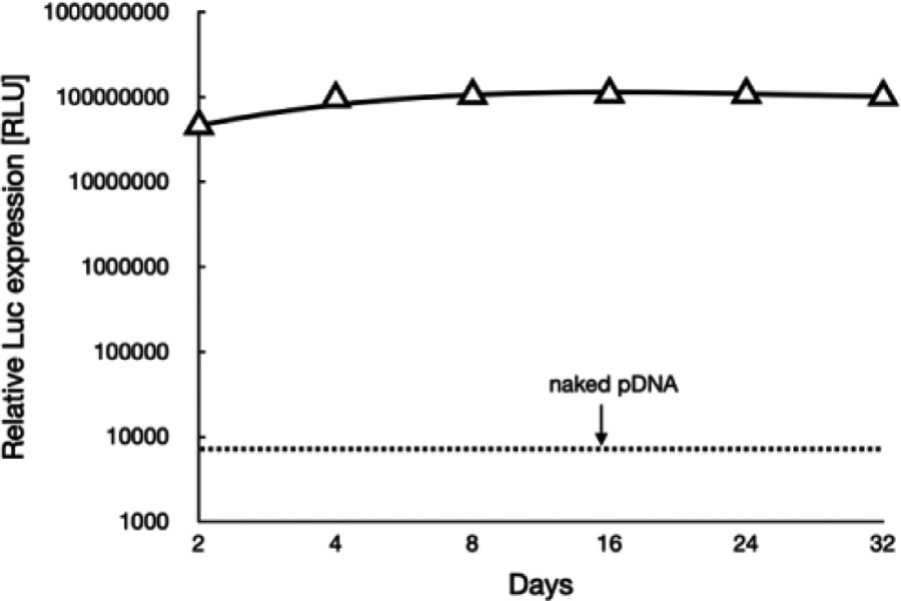
Transgene expression of Gaussia luciferase after transfection to HUVEC spheroids using synthetic gene delivery system from RGD-PEG-PLys(thiol).

Herein, the cellular uptake and gene expression efficiencies were evaluated in HUVECs. Note that HUVECs are angiogenic endothelial cells that widely used as the model cells representative for vascular endothelial cells. To start up, the cellular uptake efficiencies of a class of polyplex micelles, including PEG-PLys/pDNA, PEG-PLys(thiol)/pDNA and RGD-PEG-PLys(thiol)/pDNA, were evaluated in HUVECs by flow cytometry and CLSM measurements (Fig. 4A). Overall, cell uptake appeared for all the polyplex micelles, implying the importance of fabrication of nanoscaled DNA condensates in pursuit of cell internalization. In details, limited cellular uptake efficiency was confirmed for PEG-PLys/pDNA (Fig. 4A). Especially, no additional cell uptake was observed for PEG-PLys/pDNA after 4 h transfection (Fig. 4A), which should be as a result of its poor colloidal stabilities in the biological milieu. Presumably, PEG-PLys/pDNA was susceptible to structural dissociation in the extracellular conditions, and structural disassembly occurred within the initial 4 h incubation (Fig. 3). Namely, minimal nanoscaled self-assemblies remain after 4 h incubation, therefore very limited cellular uptake efficiency could be expected despite extended incubation. On the contrary, consistent cellular uptake was observed for PEG-PLys(thiol)/pDNA, suggesting the validity of our disulfide strategy in enhancing colloidal stabilities to seek improved cellular uptake efficiency. To our interests, RGD-PEG-PLys(thiol) mediated highest level of cellular uptake (Fig. 4A). A plausible reason for this appreciable cellular uptake efficiency from RGD-PEG-PLys(thiol)/pDNA should be attributable to the specific affinity of RGD and its receptors which are overexpressed across a number of angiogenic cells (including HUVECs), consequently specific interactions between RGD ligand and receptors affording favorable cellular endocytosis activities into the angiogenic HUVECs. In consistent with cellular uptake results, the gene expression efficiency appeared to follow a similar trend (Fig. 4B), wherein the pDNA construct from RGD-PEG-PLys(thiol) presented highest transfection efficiency, markedly higher than the self-assemblies from PEG-PLys(thiol) and PEG-PLys. Hence, the self-assemblies from RGD-PEG-PLys(thiol) was ultimately employed for encapsulation of anti-VEGF gene for treatment of CNV. It is also should be noted that long-term gene expression was confirmed from our proposed genomic therapeutic (Fig. 5). The proposed genomic construct encapsulating soluble Gaussia luciferase (Gluc)-encoding pDNA was attempted for transfection with HUVEC-based spheroids, and the extracellular expression of Gluc was quantified up to 32 d post transfection. As shown in Fig. 5, consistent gene expression was observed, and no significant decline was confirmed despite extended incubation. This long-term gene expression profile is apparently favorable to the ultimate therapeutic outcomes, namely the therapeutic proteins from our proposed gene therapeutic strategy were believed to conduce to potent and enduring anti-angiogenic microenvironment to suppress the pathogenesis.

### 3.5 Remarkable *in vivo* expression of anti-angiogenic proteins and potent suppression on CNV

**Fig. 6:**
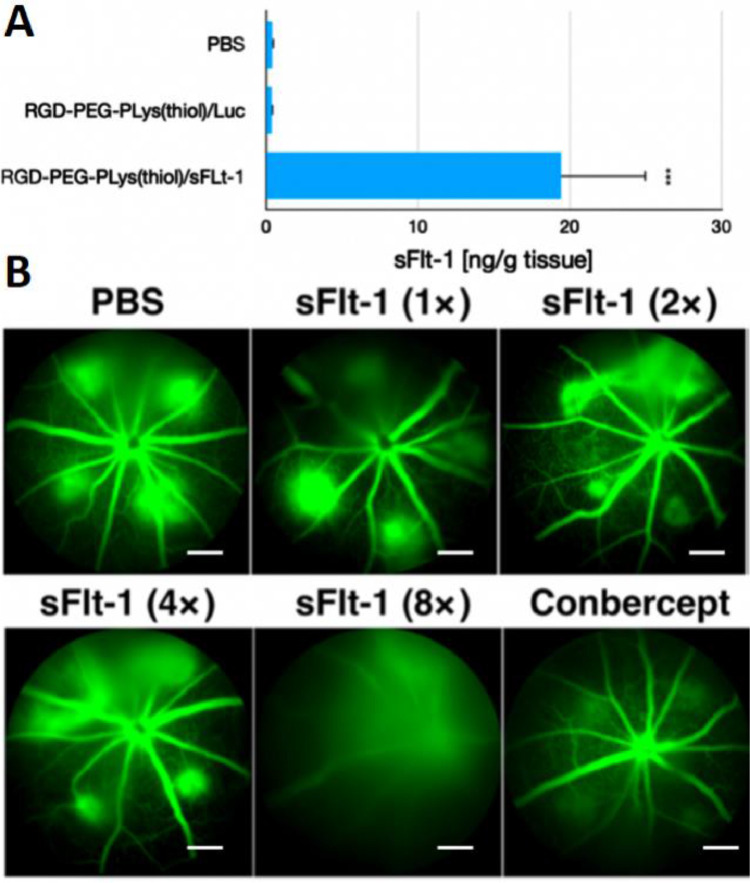
Therapeutic assessment on our proposed anti-angiogenic therapeutics composed of RGD-PEG-PLys(thiol). (A) Quantification of the expressed sFlt-1 by ELISA. (B) Insight into the CNV leakage by ocular fluorescence angiography on Day 7 post treatment of synthetic gene delivery system from RGD-PEG-PLys(thiol) (<1× > assigned to be 0.1 µg sFlt-1 pDNA). Note that intraperitoneal administration of fluorescein sodium was schemed prior to observation, scale bar: 200 µm.

**Fig. 7:**
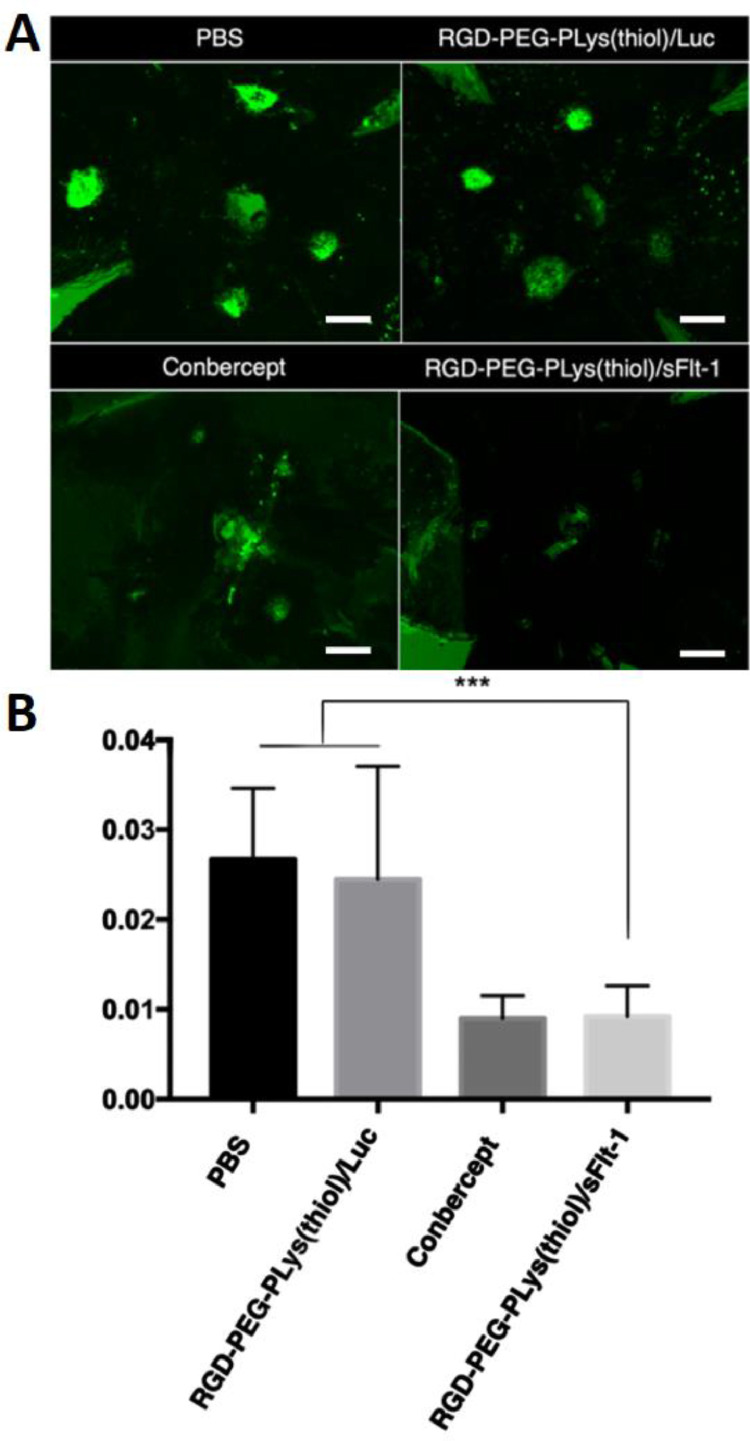
Evaluation on choroidal vascular densities upon diverse treatment. (A) the choroidal flatmount images to illustrate neovasculatures, scale bar: 100 µm; (B) the quantified densities of neovasculature were summarized into bar graph.

With the aim of suppressing CNV, an anti-VEGF gene (plasmid DNA) encoding soluble fms-related tyrosine kinase 1 (sFlt-1), was attempted as the genomic payloads for construction of gene delivery systems. In view that VEGF plays a fundamental role in promoting neovascularization and edema in wAMD, disruption of VEGF signaling should serve as a rational approach to interfere the progression of CNV. Herein, CNV were induced according to our previously established laser photocoagulation protocol. At 24 h post photocoagulation, CNV was confirmed by fluorescence angiography. To explore the therapeutic efficacy of our constructed anti-VEGF gene delivery systems, varied dosages of RGD-PEG-PLys(thiol)-based self-assemblies containing anti-VEGF pDNA (sFlt-1) was administered *via* intravitreous injection. The expression levels of the therapeutic payloads of sFlt-1 pDNA were evaluated in CNV regions by ELISA at 48 h post dosage. As shown in Fig. 6A, markedly sFlt-1 expression was confirmed from our proposed gene delivery systems from RGD-PEG-PLys(thiol), which is envisioned to endow anti-VEGF microenvironment in suppressing angiogenesis. Herein, the therapeutic efficacy was assessed by fluorescence angiography on Day 7 post treatment. As shown in Fig. 6B, aggressive neovasculature was observed for the control PBS group. On the contrary, the commercial VEGF antibody of Conbercept appeared to exert potent inhibition on progression of neovascularization, as evidenced by reduced angiogenic blood vessels. Noteworthy is the efficacy of our proposed gene delivery systems containing sFlt-1, which exhibited significant suppression in the growth of neovasculature (Fig. 6B). Close examination identified the suppression activities followed distinct dosage-dependent manner, and significant eradiation of neovasculature was achieved at dosage of 0.8 µg sFlt-1 pDNA. Furthermore, aiming for quantitatively estimation of the therapeutic efficacies, the vascular densities of the CNV lesions were quantified by calculating the positive areas of choroidal blood vessels post choroidal flatmount and immunostaining. Significant lowered CNV areas were confirmed for the sample of RGD-PEG-PLys(thiol)/sFlt-1 (Fig. 7). It should be noted that, there was no significant difference between the PBS control group RGD-PEG-PLys(thiol)/Luc (containing non-therapeutic Luc gene), suggesting the obtained therapeutic efficacies as a result of expression of the loaded anti-angiogenic sFlt-1. Presumably, RGD-PEG-PLys(thiol)/sFlt-1 due to its specific affinity to the angiogenic endothelial cells, could preferentially accumulate in the blood vessels. Furthermore, the proposed redox-responsive motif of the proposed RGD-PEG-PLys(thiol)/sFlt-1 self-assembly allowed efficient gene expression in the targeted endothelial cells. The expressed sFlt-1, once secreted from the cells, manage to diffuse around and readily trap the angiogenic VEGF cytokines, ultimately contributing to inhibited neovasculature. Noteworthy is the therapeutic efficacy of our proposed gene delivery systems based on single dosage comparable to the most potent antibody-based therapeutics (Conbercept), validating its clinical potential in treatment of wAMD. Hence, the prosperous potential of anti-angiogenic strategy in treatment of wAMD was verified based on intravitreous injection of gene delivery systems encapsulating sFlt-1 gene. Aside from the therapeutic efficacy, appreciable safety profile was also confirmed for our constructed gene delivery systems, as evidenced by their excellent biocompatibilities. As shown in Fig. S8, negligible cytotoxicity was confirmed for the self-assemblies of RGD-PEG-PLys(thiol)/pDNA, meanwhile, minimal hemolysis was also verified when RGD-PEG-PLys(thiol)/pDNA was incubated with defibrinated sheep red blood cells. At 6 h post incubation, the hemolysis rates of RBCs were verified to be rather negligible (merely 2% even at a high concentration up to 0.2 mg/ml, Fig. S9), in stark contrast to the hemolysis rates of RBCs under incubation with amphiphiles of Tween80 to be 20.1% and 72.8% at the concentration of 0.25 and 1 mg/ml). Together with appreciable therapeutic efficacies, our proposed anti-angiogenic gene delivery system of RGD-PEG-PLys(thiol)/pDNA illustrates an intriguing tool for clinical persistent inhibition of CNV.

## 4 Conclusions

The rising population suffering from CNV encouraged tremendous efforts in development of potent therapeutics to tackle with this global leading cause for irreversible blindness. To manage this formidable disease, we are motivated to construct anti-angiogenic genomic therapeutics to verify the feasibility of gene therapy in treatment of CNV. In the present study, synthetic gene delivery system was rationally tailored with multifunctional motifs, including active targeting function to the angiogenic endothelial cells, stimuli-responsive stabilities to release the DNA payloads selectively in the cell interiors, which consequently contributed to efficient gene expression at the targeted cells. Note that the constructed delivery system encapsulating anti-angiogenic gene was determined to accomplish potent CNV suppression. Therefore, our proposed gene delivery system implied tremendous potential of gene therapy as an alternative approach for treatment of CNV. Considering the versatile genomic tools available in gene therapy, for instance, Crispr/Cas gene editing or RNA interference, our proposed gene delivery system could be further exploited to find broad utilities in achieving appropriate therapeutic consequences in treatment of intractable ocular diseases.

## Conflicts of interest

The authors declare no competing financial interest.

## Appendix Supplementary materials

Image, application 1
