# Supplementary material for: Synthetic anti-angiogenic genomic therapeutics for treatment of neovascular age-related macular degeneration
Source: Asian J Pharm Sci. 2021 May 7;16(5):623–32. doi: 10.1016/j.ajps.2021.04.001 (PMC8609386; doi:10.1016/j.ajps.2021.04.001)
Supplement: Supplementary file 1 [file mmc1.pdf]

Supporting Information

Synthetic Anti-Angiogenic Genomic Therapeutics for Treatment of Neovascular Age-Related Macular Degeneration

Jing Wang[1#], Xiang Shi[1#], Qiyu Bo[1], Hong Wang[1], Fang Wei[2], Jun Liu[5], Hao Wang[6], Liuwei Zhang[6], Yan Qi[7], Zhen Li[7], Qixian Chen[6*], Xiaodong Sun[1,2,3,4**]

1. Department of Ophthalmology, Shanghai General Hospital, Shanghai Jiao Tong University School of Medicine, No. 100 Haining Road Shanghai 200080, Shanghai, China

2. Shanghai Key Laboratory of Ocular Fundus Diseases, Shanghai General Hospital, Shanghai Jiao Tong University School of Medicine, No. 100 Haining Road Shanghai 200080, Shanghai, China

3. Shanghai Engineering Center for Visual Science and Photomedicine, Shanghai General Hospital, Shanghai Jiao Tong University School of Medicine, No. 100 Haining Road Shanghai 200080, Shanghai, China

4. National Clinical Research Center for Eye Diseases, Shanghai General Hospital, Shanghai Jiao Tong University School of Medicine, No. 100 Haining Road Shanghai 200080, Shanghai, China

5. Ningbo Hygeia Medical Technology Co., Ltd., No. 6 Jinyuan Road, High-Tech Zone, Ningbo 315201, China

6. School of Bioengineering, Dalian University of Technology, No. 2 Linggong Road, Dalian 116024, China

7. College of Pharmacy, Dalian Medical University, No. 9 West Section Lvshun South Road, Dalian 116044, China

*All correspondence should be addressed to Q. C. (E-mail: qixian@dlut.edu.cn) and X. S. (xdsun@sjtu.edu.cn)

Synthesis of PEG-PLys. Block copolymer of PEG-PLys was synthesized according to a ring-opening polymerization approach according to the previous report [S1]. In brief, monomer of Lys(TFA)-NCA was polymerized from initiation of the ω-NH_2 terminal group of methoxy (MeO)-PEG in *N,N*-dimethylformamide (DMF). The molecular weight distribution (M_w/M_n) of the yielded PEG-PLys(TFA) was determined to be 1.05 from gel permeation chromatography (GPC) equipped with TOSOH HLC-8220 calibrated based on varying M_w of commercial PEG standards. Furthermore, MeO-PEG-PLys(TFA) was dissolved in methanol containing 1 N NaOH with the aim of remove protective TFA groups at 30 °C for overnight reaction.

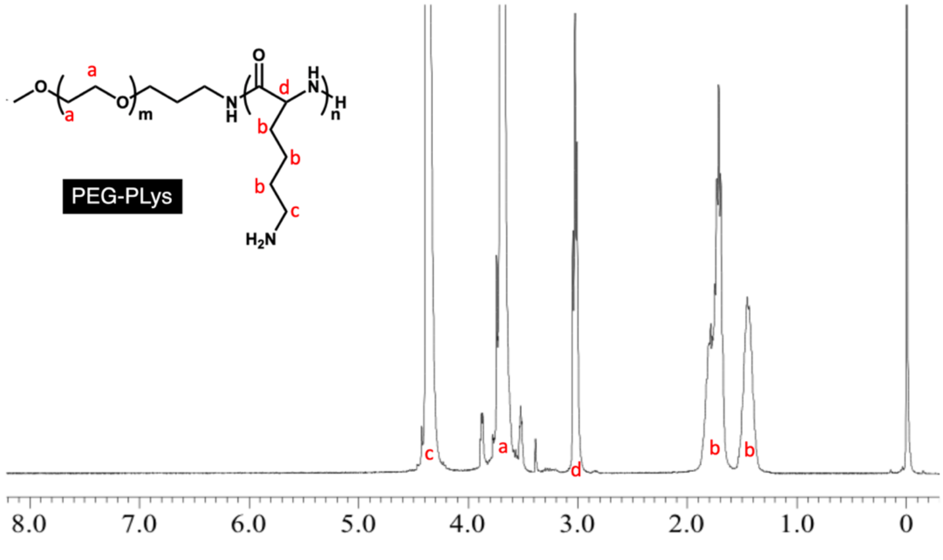

Fig. S1 ^{1}H-NMR spectra of PEG-PLys in D_2O at 80 °C.

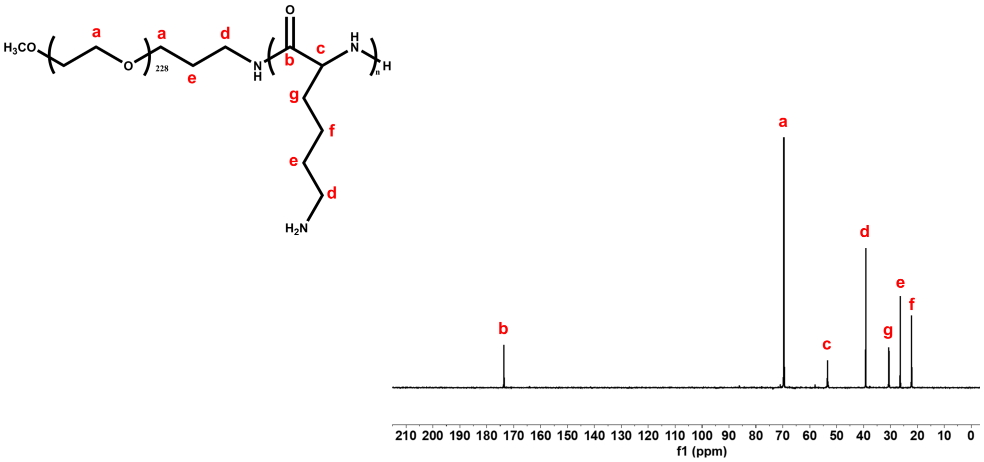

Fig. S2 C-NMR spectra of PEG-PLys.

Synthesis of acetal-PEG-PLys. Following a similar synthetic procedure, acetal-PEG-PLys was also synthesized.

Synthesis of thiolated PEG-PLys [PEG-PLys(thiol]. Thiolated PEG-PLys(thiol) were prepared by introducing pyridyldithiopropionyl (PDP) groups into the side chain of lysine units of the PLys segment of PEG-PLys using the heterobifunctional reagent *N*-succinimidyl 3-(2-pyridyldithio) propionate (SPDP). In brief, PEG-PLys was dissolved in *N*-methyl-2-pyrrolidone (NMP) supplemented with 5 wt % LiCl and reacted with predefined concentration of SPDP pre-dissolved in NMP containing *N*, *N*-diisopropylethylamine (10 mol excess against SPDP) at room temperature. After 4 h reaction, the crude product was purified by precipitation into diethyl ether. Furthermore, the precipitated product was dissolved in 0.01 N HCl, dialyzed against the distilled water, and lyophilized to obtain PEG-PLys(PDP).

Synthesis of thiolated acetal-PEG-PLys [acetal-PEG-PLys(thiol)]. Block polymer acetal-PEG-PLys(thiol) was synthesized according to a similar synthetic procedure, which was further used for cRGD ligand conjugation.

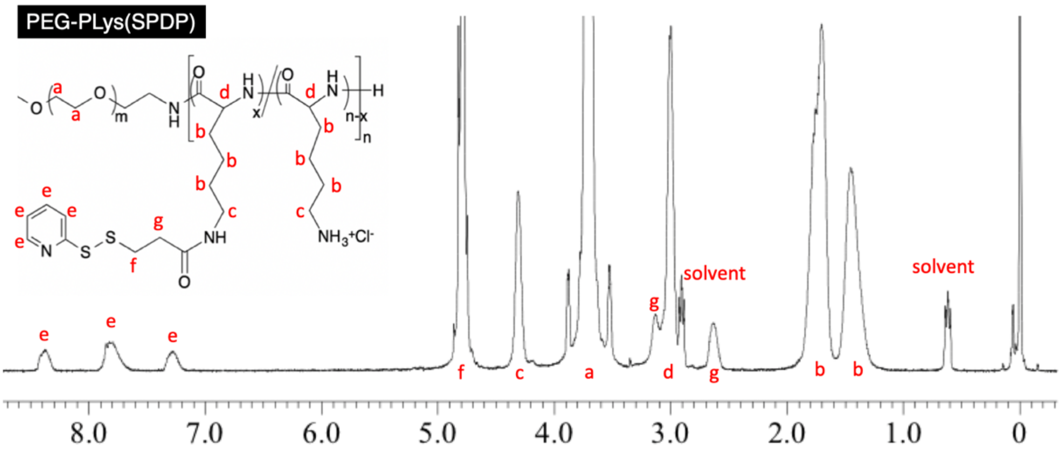

Fig. S3 ^{1}H-NMR spectra of acetal-PEG-PLys(PDP) in D_2O at 80 °C.

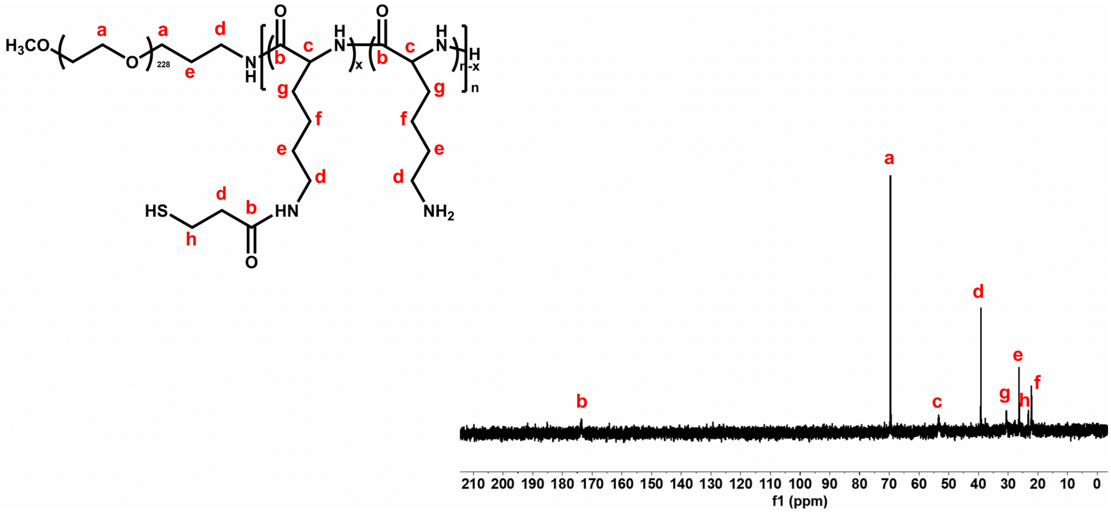

Fig. S4 C-NMR spectra of PEG-PLys(thiol).

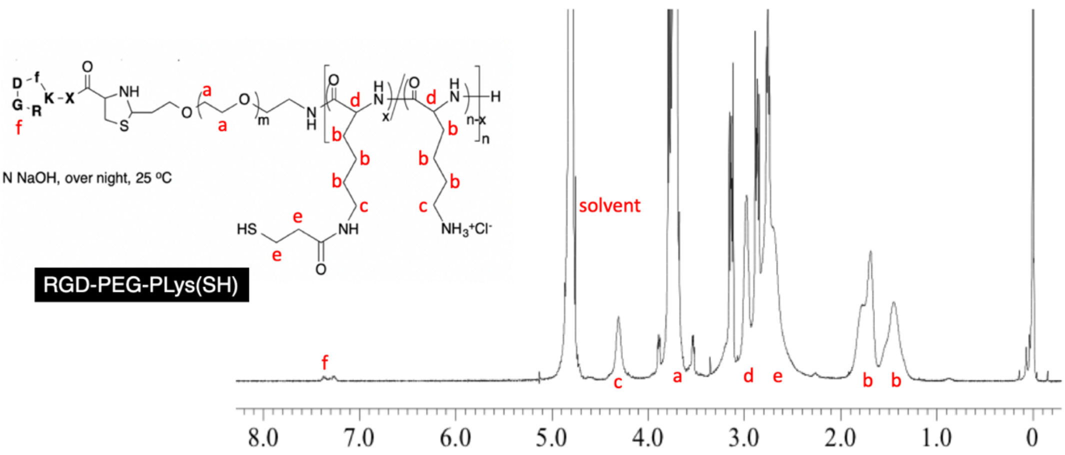

Fig. S5 ^{1}H-NMR spectra of RGD-PEG-PLys(PDP) in D_2O at 80 °C.

Synthesis of RGD-PEG-PLys(thiol). The cyclo[RGDfK(C-ε-Acp)] (cRGD) peptide ligand was attached onto the α-terminal of acetal-PEG-PLys(thiol) through formation of a thiazolidine ring between the *N*-terminal cysteine of the cRGD peptide and the aldehyde group from acetal-PEG-PLys following incubation at acidic pH (pH = 2) [S2]. In brief, acetal-PEG-PLys(thiol) was dissolved in 10 mM HEPES buffer (pH 7.4) and dialyzed against 0.01 N HCl (pH 2.0) to yield aldehyde from the acetal group. The pH of the dialyzed solution was adjusted to 2 by 0.01 N NaOH, following which the pre-DTT-treated cRGD solution was added dropwise while stirring. After stirring overnight at 25 °C, the polymer solution was dialyzed against HEPES with 150 mM NaCl, followed by distilled water. Eventually, the solution was collected and lyophilized to obtain cRGD-PEG-PLys(thiol). The percentage of cRGD conjugation was determined by the peak intensity ratio of benzyl protons (D-Phenyl alanine, f: D-Phe; δ = 7.3–7.4 ppm) of the cRGD peptide to the methylene protons of PEG (δ = 3.7 ppm) from the ^{1}H-NMR spectra, and the conjugation ratio of cRGD was calculated to be 95%.

GPC. cRGD-PEG-PLys(thiol) was dissolved in PBS (10 mM, pH 7.4) at a concentration of 1 mg/mL, followed by supplementation with glutathione (GSH)-containing PBS solution to possess the final GSH concentration of 50 mM and final polymer concentration of 0.5 mg/mL. The reaction solution was incubated at 37 °C for 6 h and transferred for aqueous GPC measurement. Note that the eluent carrier was PBS (10 mM, pH 7.4) containing 150 mM NaCl, which also have the GSH concentration of 50 mM.

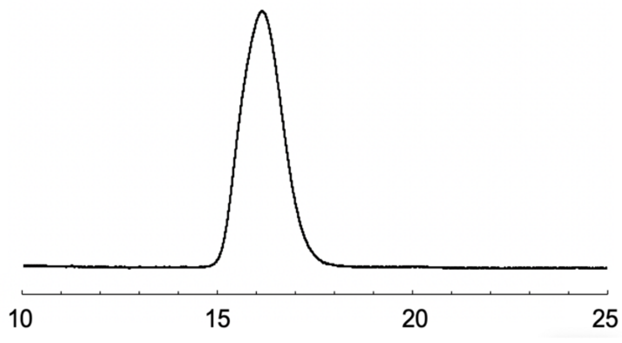

Fig. S6 GPC trace for copolymeric RGD-PEG-PLys(thiol).

Gel electrophoresis.

The complexations of pDNA with copolymeric RGD-PEG-PLys(thiol) at varied N/P ratio were confirmed by agarose gel retardation analysis. In the gel retardation analysis, each sample was prepared by the dilution of the complex solutions to the concentration of 15 mg pDNA/L. 0.020 mL of each sample (300 ng pDNA) with a loading buffer was then electrophoresed at 50 V for 2 h on a 0.9 wt% agarose gel in 3.3 mM Tris–acetic acid buffer containing 1.7 mM sodium acetate. The migrated pDNA was visualized by soaking the gel in distilled water containing EtBr (0.5 mg/L).

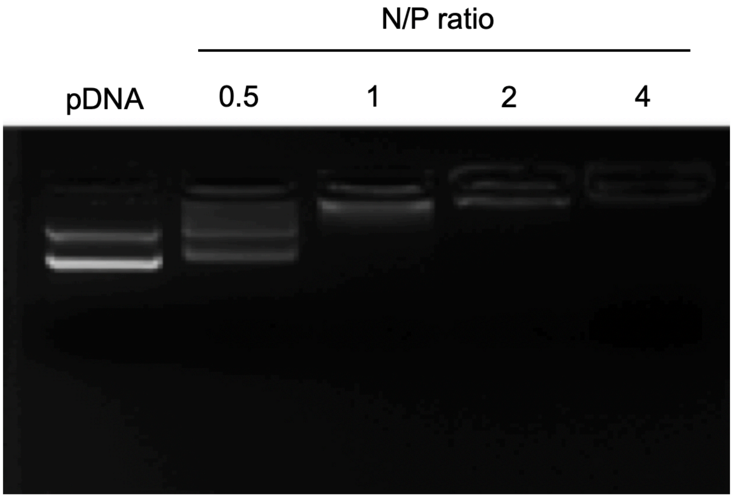

Fig. S7 Gel electrophoresis for complexation of RGD-PEG-PLys(thiol) and pDNA at varied N/P ratios.

Cytotoxicity. HUVECs were plated onto 24-well culture dishes (20,000 cells/well) in 400 µl of cell culture containing 10% FBS and 1% antibiotics (penicillin and streptomycin) and incubated in a humidified atmosphere with 5% CO_2 at 37 °C. After 24 h of incubation, the medium was replaced with 400 µl of fresh medium, followed by the addition of pDNA formulation solutions. After 24 h incubation, the medium was replaced with fresh medium, followed by another 24 h of incubation. The cells were washed three times with ice-cold PBS, followed by the addition of 200 µl of fresh medium. Cell viability was assessed on the

basis of 2-(2-methoxy-4-nitrophenyl)-3-(4-nitrophenyl)- 5-(2,4-disulfophenyl)-*2H*-tetrazolium (WST-8) reduction to WST-8 formazan by the dehydrogenase activity of viable cells using the Cell Counting Kit-8 (CCK-8) (Dojindo, Kumamoto, Japan) according to manufacturer's instructions. In brief, 20 µl of the CCK-8 reagent was added to each well and allowed to develop orange-colored WST-8 formazan for 2 h. The UV absorbance of WST-8 formazan in each well was quantified at 450 nm using a microplate reader (Model 680, Bio-Rad, UK). The cell toxicity was expressed as the percentage of cell viability normalized against control cells treated with 10 mM HEPES (pH 7.4) (n = 4).

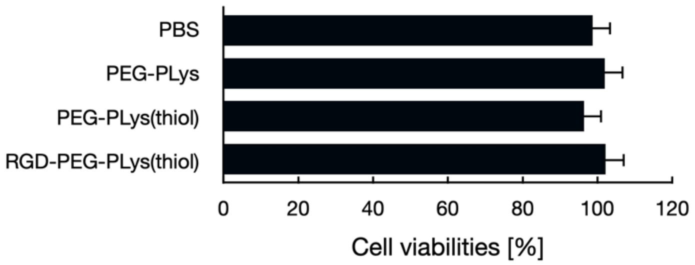

Fig. S8 Cell viabilities of HUVECs in presence of synthetic gene delivery systems (0.1 mg pDNA /mL).

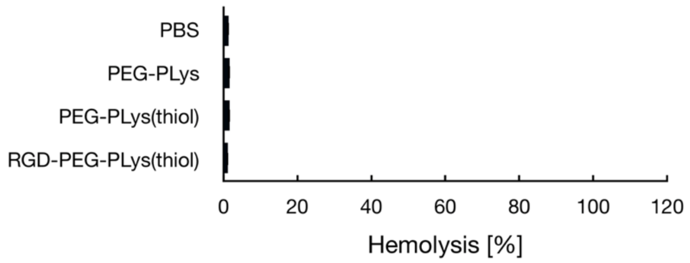

Fig. S9 Hemolytic activities of a class of synthetic gene delivery system under incubation with defibrinated sheep red blood cells at 37 °C.

Genomic sequence of pDNA.

a) Luciferase-encoding pDNA (pCAG-Luc)

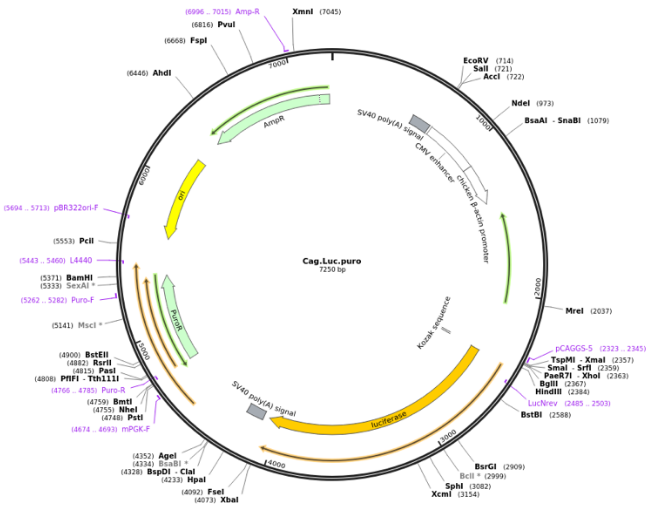

b）Red fluorescence protein-encoding pDNA (pCAGGs-RFP)

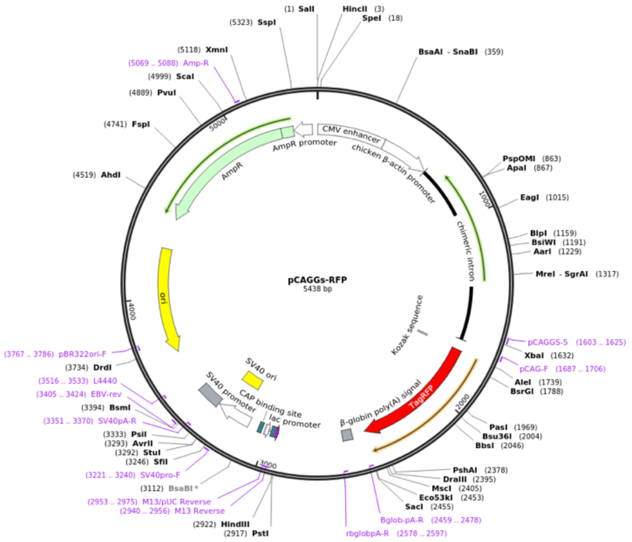

c) Soluble Flt1-encoding pDNA (pCMV-sFlt1)

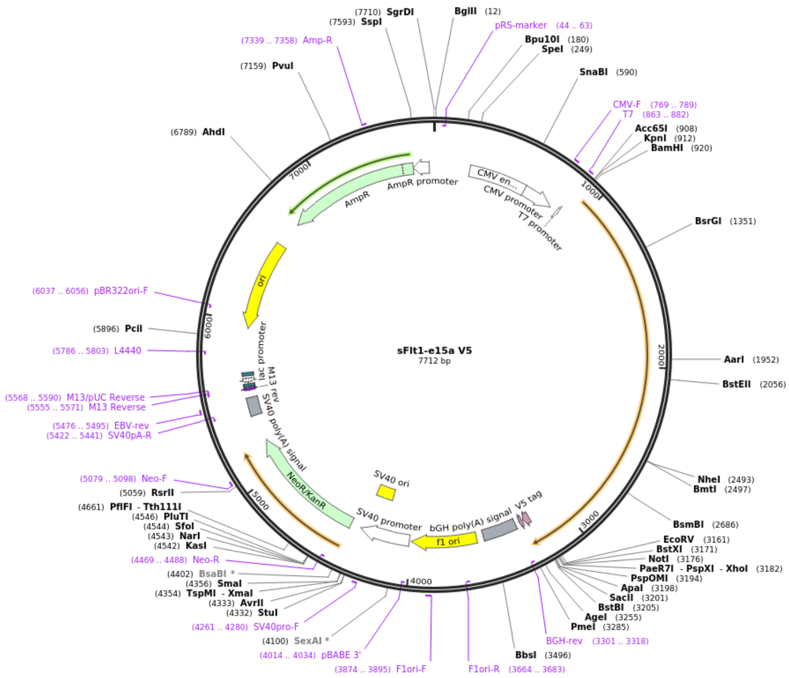

References

[S1] Chen, Q., Qi, R., Chen, X., Yang, X., Huang, X., Xiao, H., Wang, X., and Dong, W. (2016) Polymeric Nanostructure Compiled with Multifunctional Components To Exert Tumor-Targeted Delivery of Antiangiogenic Gene for Tumor Growth Suppression. *Acs Appl. Mater. Interfaces* 8, 24404-24414.

[S2] Ge, Z., Chen, Q., Osada, K., Liu, X., Tockary, T. A., Uchida, S., Dirisala, A., Ishii, T., Nomoto, T., Toh, K., Matsumoto, Y., Oba, M., Kano, M. R., Itaka, K., and Kataoka, K. (2014) Targeted gene delivery by polyplex micelles with crowded PEG palisade and cRGD moiety for systemic treatment of pancreatic tumors. *Biomaterials* 35, 3416-3426.
